# *In vitro* substrate reduction, chaperone and immunomodulation treatments reduce heparan sulfate in mucolipidosis III human fibroblasts

**DOI:** 10.1590/1678-4685-GMB-2023-0117

**Published:** 2023-12-04

**Authors:** Fernanda Sperb-Ludwig, Nataniel Floriano Ludwig, Gustavo Mottin Rizowy, Renata Voltolini Velho, Ida Vanessa Doederlein Schwartz

**Affiliations:** 1 Hospital de Clínicas de Porto Alegre, Laboratório BRAIN, Porto Alegre, RS, Brazil.; 2 Universidade Federal do Rio Grande do Sul, Programa de Pós-Graduação em Genética e Biologia Molecular, Porto Alegre, RS, Brazil.; 3 Endometriosis Research Charité, Department of Gynecology Charité with Center of Oncological Surgery, Virchow-Klinikum, Berlin, Germany.; 4 Hospital de Clínicas de Porto Alegre, Serviço de Genética Médica, Porto Alegre, RS, Brazil.

**Keywords:** Substrate reduction therapy, Mucolipidosis II and III, Genistein, Miglustat, Thalidomide

## Abstract

Mucolipidosis II and III (MLII and MLIII) are autosomal recessive diseases caused by pathogenic variants in *GNPTAB* and *GNPTG* genes that lead to defects in GlcNAc-1-phosphotransferase. This enzyme adds mannose 6-phosphate residues to lysosomal hydrolases, which allows enzymes to enter lysosomes. Defective GlcNAc-1-phosphotransferase causes substrate accumulation and inflammation. These diseases have no treatment, and we hypothesized that the use of substrate reduction therapy and immunomodulation may be beneficial at the cell level and as a future therapeutic approach. Fibroblasts from two patients with MLIII alpha/beta and 2 patients with MLIII gamma as well as from one healthy control were treated with 10 µM miglustat, 20 µM genistein, and 20 µM thalidomide independently. ELISA assay and confocal immunofluorescence microscopy were used to evaluate the presence of heparan sulfate (HS) and the impact on substrate accumulation. ELISA assay showed HS reduction in all patients with the different treatments used (p=0.05). HS reduction was also observed by immunofluorescence microscopy. Our study produced encouraging results, since the reduction in substrate accumulation, even partial, may offer benefits to the phenotype of patients with inborn errors of metabolism.

## Introduction

Mucolipidosis type II (MLII, MIM#252500) and type III (MLIII alpha/beta, MIM#252600; MLIII gamma, MIM#252605) are rare autosomal recessive lysosomal storage disorders caused by deficient activity of N-acetylglucosamine-1-phosphotransferase (GlcNAc-1-phosphotransferase; EC 2.7.8.17). This enzyme is responsible for catalyzing the addition of mannose 6-phosphate residues to lysosomal hydrolases and plays an essential role in receptor-mediated transport to the endosomal and prelysosomal compartments. A breakdown in this recognition signal can lead to incorrect targeting of lysosomal enzymes. Patients with MLII and MLIII have defective lysosomal enzymes in several types of cells with substrate accumulation and increased enzyme activity in their extracellular fluids, such as serum and plasma (Hasilik and Neufeld, 1980; Hasilik and Von Figura, 1981; Reitman et al., 1981; Kornfeld and Sly, 2001).

The clinical manifestations of MLII and MLIII are multisystemic and cover a wide spectrum, which is reflected in the high variability among affected individuals (Hasilik and Neufeld, 1980; Hasilik and Von Figura, 1981; Reitman et al., 1981; Kornfeld and Sly, 2001; Braulke and Bonifacino, 2009). In the absence of useful genetic and biochemical markers, the distinction between MLII and MLIII can be made using clinical criteria (Tiede et al., 2006). 

MLII is caused by mutations in the *GNPTAB* gene, and affected individuals experience an early onset of symptoms and high disease severity with a progressive course generally leading to death in childhood (Kornfeld and Sly, 2001; Braulke and Bonifacino, 2009; Alegra et al., 2019). MLIII alpha/beta is also caused by *GNPTAB* mutations with onset of symptoms in childhood. However, disease progression is slower, with death generally occurring in adulthood. MLIII gamma is caused by mutations in the *GNPTG* gene and, although it resembles MLIII alpha/beta, its natural history is still underreported in the literature (Okada et al., 1985; Meikle et al., 1999, Alegra *et al.*, 2019). 

There is no cure for MLII or MLIII. Currently available therapies for MLII and MLIII, as well as for several other lysosomal diseases, are limited to supportive care and treatment of complications, with the use of palliative life-sustaining measures (Alegra et al., 2019). Remarkable advances in the ability to treat lysosomal diseases have occurred in recent decades due to the development of new therapies based on new molecules and drugs, innovative techniques, and increased knowledge of genetic diseases.

In this context, we hypothesized that the use of substrate reduction therapy and immunomodulation may be a therapeutic approach to ameliorate cell clearance and homeostasis, thus reducing symptoms of MLIII. Genistein is an isoflavone that acts as a broad-spectrum protein tyrosine kinase inhibitor, acting on several growth factor receptors, which are important for proteoglycan synthesis (Piotrowska *et al.*, 2008). N-butyldeoxynojirimycin (miglustat) is an inhibitor of glucosylceramide synthase (GlcCerS), thus reducing the synthesis of glucocerebrosides and gangliosides and acting as a chaperone in different diseases (Elliot-Smith *et al.*, 2008; Khan and Tomatsu, 2020; Kavanagh and Pastores, 2021). Thalidomide is a drug that acts by regulating the immune system and reducing inflammation (Simonaro, 2016).

To test this hypothesis, we performed *in vitro* treatments on fibroblasts from patients with MLIII to assess heparan sulfate (HS) substrate accumulation as an initial response.

## Material and Methods

Skin fibroblasts from four patients with MLIII and 1 healthy control were analyzed. The cultivated skin fibroblasts were treated independently with 20 µM genistein (Wako), 10 µM miglustat (Zavesca®), or 20 µM thalidomide (Fundação Ezequiel Dias) in Dulbecco’s modified Eagle’s medium (DMEM) low-glucose medium (Gibco) supplemented with 10% fetal bovine serum (Gibco) and 1% penicillin/streptomycin (Gibco) in a 5% CO_2_ atmosphere at 37 °C for 5 days. 

Confocal immunofluorescence microscopy was performed to evaluate the presence of HS using anti-HS monoclonal antibody (1:100; 10E4; Amsbio), followed by staining with Alexa Fluor 488 (1:500; Molecular Probes) and DAPI (Molecular Probes) to visualize the nucleus.

HS accumulation was also detected by using the Human Heparan Sulfate Proteoglycan 2 ELISA Kit (Abbexa), which was performed with total proteins according to the manufacturer’s instructions. Statistical analysis was performed with the independent-samples Mann-Whitney U test in SPSS, version 16.0.

The study was approved by the Ethics Committee of the Hospital de Clínicas de Porto Alegre, Brazil, number 12-0276.

## Results

Four patients with MLIII were analyzed: patient 1 - c.[328G>T];[328G>T] (*GNPTG*; MLIII gamma); patient 2 - c.[1931C>T];[3668_3670delCTA] (*GNPTAB*; MLIII alpha/beta); patient 3 - c.[244_247dupGAGT];[328G>T] (*GNPTG*; MLIII gamma); and patient 4 - c.[3503_3504delCT];[?] (*GNPTAB*; MLIII alpha/beta). 

**Figure 1-:**
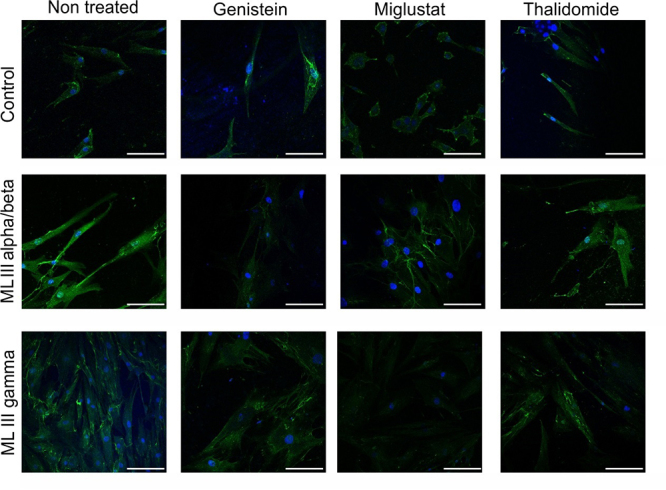
Heparan sulfate (HS) accumulation in fibroblasts from a healthy control and from patient 2 (MLIII alpha/beta) and patient 3 (MLIII gamma) receiving different treatments with genistein, miglustat, and thalidomide or untreated. Green is HS and blue is DAPI/nucleus

Confocal immunofluorescence microscopy showed HS accumulation in the cytosol and surface of fibroblasts from the healthy control and patients with MLIII, with a perceptibly higher fluorescence intensity in patients (Figure 1). HS accumulation was higher in untreated cells from patients with MLIII alpha/beta and MLIII gamma than in their cells treated with genistein, miglustat, and thalidomide (Figure 1).

**Figure 2-:**
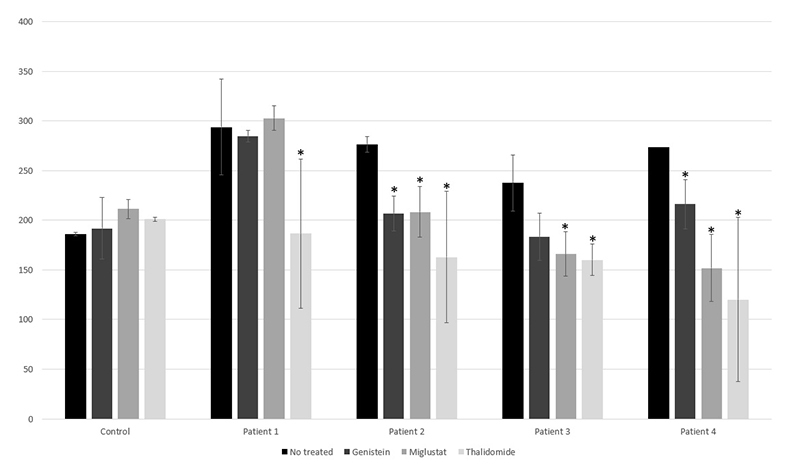
Intracellular heparan sulfate (HS) amount (μg HS/mg protein) in untreated and treated cells. All experiments were conducted in triplicate. *Statistically significant reduction in HS accumulation compared with untreated (p=0.05)

The quantification of intracellular HS accumulation for the different treatments is shown in Figure 2. When comparing the patients individually for the accumulated amount of HS in untreated vs treated cells, substrate reduction was observed in patient 1 for thalidomide (p=0.05), in patient 2 for genistein (p=0.05), miglustat (p=0.05), and thalidomide (p=0.05), in patient 3 for miglustat (p=0.05) and thalidomide (p=0.05), and in patient 4 for genistein (p=0.05), miglustat (p=0.05), and thalidomide (p=0.05). Confocal microscopy in patients 2 and 3 showed an HS signal intensity consistent with the accumulation quantified by ELISA (Figure 1). 

Comparing the treatment groups with the control, the results were reduction in substrate accumulation for genistein (p=0.049), miglustat (p=0.044), and thalidomide (p=0.01).

## Discussion

Substrate accumulation affects the endosomal/lysosomal targeting system, leading to imbalance in cell homeostasis, changes in metabolic pathways, activation of autophagic processes, and cell death. In this context, substrate reduction therapy and regulation of inflammatory and immune processes would be an interesting approach to treat storage diseases (Walkley, 2009). Substrate reduction therapy represents an important approach in the treatment of lysosomal diseases. The concept of this therapeutic principle is to reduce the amount of storage material rather than increase the activity of degrading enzymes, thus helping to redress the imbalance between the rate of biosynthesis and the rate of catabolism (Sato et al., 2003; Cox, 2005). 

Immunomodulation and control of inflammation in metabolic diseases may be an option in the attempt to improve the phenotype of genetic diseases, since they could attenuate the pathophysiological cell abnormalities involved in the process. Storage of macromolecules activates inflammatory pathways, resulting in local and systemic inflammation caused by substrate storage or general lysosomal dysfunction such as defective autophagy (Simonaro, 2016).

Otomo et al. (2012) showed in MLII cells that HS accumulated in the cytosol and cell surface, and the same result was obtained in the present study for patients with MLIII. The presence of HS had never been demonstrated in cells from patients with MLIII to date, nor had they been evaluated with different forms of treatment. 

The amount of HS accumulated in MLIII cells is proportionally smaller than that in MLII cells (Otomo et al., 2012), as expected from disease severity. MLIII shows accumulation of saccharides, proteins, cholesterol, phospholipids, sphingolipids, and glycosaminoglycans in the cells. The treatment potential of genistein and miglustat lies in their capacity to act precisely on the synthesis of glycosaminoglycans and glycosphingolipids, thus reducing substrates, in addition to the chaperone effect of miglustat (Elliot-Smith *et al.*, 2008). 

In the mechanism of action of genistein, the epidermal growth factor (EGF) binds to its transmembrane receptor triggering a tyrosine kinase signaling cascade that results in the regulation of transcription factor activity. This tyrosine-specific protein kinase activity of the EGF receptor has its phosphorylation inhibited by genistein, which leads to a reduction in the synthesis of glycosaminoglycans (Piotrowska *et al.*, 2008). Miglustat, as well as its related compounds, can inhibit GlcCerS and, therefore, reduce the synthesis of glucocerebrosides and gangliosides, such as ganglioside GM3, substrates also accumulated in MLIII (Boutry et al., 2018; Khan and Tomatsu, 2020). Miglustat has been used clinically for the purpose of reducing substrate and as a chaperone in patients with Gaucher disease and Niemann-Pick type C disease (Patterson et al., 2007; Pineda et al., 2018; Breiden and Sandhoff, 2020). The action observed in MLIII HS may be the result of lower glycosphingolipid accumulation. This is reflected in the cellular response mechanisms that protect cells from the consequences of lysosomal damage. This process ensures lysosomal quality control and clearance, by processes such as autophagy and mitophagy, and therefore cell homeostasis (Papadopoulos and Meyer, 2017; Zhu et al., 2020). The effect of miglustat as a pharmacological chaperone has already been suggested in late-onset Pompe disease, GM2 gangliosidosis, and Gaucher disease (Breiden and Sandhoff, 2020; Masingue et al., 2020; Guémy and Laforêt, 2023).

Because patients with MLIII accumulate less substrate than those with MLII, the result of treatments can be even more effective in a clinical setting. The use of substrate reduction therapies in combination with enzyme replacement therapies has already been suggested, but MLII and MLIII are not amenable to enzyme replacement therapy because they affect numerous lysosomal enzymes (Coutinho et al., 2016).

Thalidomide acts by regulating the immune system and reducing inflammation. Our significant results may be explained by immunomodulation and the role of lysosomes in immunity and inflammation, regulating autophagy, controlling inflammasome activation, and regulating sphingolipid metabolism (Simonaro, 2016). Anti-inflammatory therapies have already been used in patients, cells, and animals with lysosomal storage diseases with beneficial effects (Bosch and Kielian, 2015; Seo and Kim, 2015; Yoo and Kim, 2015). Thalidomide belongs to a class of drugs that target the 3′-untranslated region (UTR) of tumor necrosis factor alpha (TNF-α) mRNA, inhibiting TNF-α production, and have multipotent and pleiotropic effects, thus being tested to treat neuroinflammation in neurodegenerative diseases (Jung et al., 2019). Although the molecular mechanisms of thalidomide are still poorly understood, new targets of thalidomide have been recently identified, such as cereblon (CRBN), a ligand-dependent substrate receptor of the E3 ubiquitin ligase complex cullin-RING ligase 4 (CRL4^CRBN^) that recognizes neosubstrates and has been used in a novel protein knockdown technology named proteolysis targeting chimeras (PROTACs) (Ito and Handa, 2020). This implies that the molecular mechanisms involving thalidomide still need to be further investigated and fully elucidated.

In the current study, the best results were obtained for patients 2 and 4, who have MLIII alpha/beta. The use of the tested drugs in humans must be further evaluated to define indications and to establish adequate doses and potential adverse effects. All tested drugs are contraindicated in pregnancy and in cases of hypersensitivity, with thalidomide being a known teratogen. Genistein is contraindicated in women with a history of cancer of the breast and reproductive tract (Asatsuma-Okumura et al., 2020; Mukund, 2020).

In conclusion, treatments with genistein, miglustat, and thalidomide appear promising for patients with MLIII and should be carefully investigated for future clinical applications. Since patients with MLIII show less accumulation of HS, the results presented here may provide a more significant improvement in the clinical presentation of the disease in patients with MLIII than MLII. A combination of treatments may offer beneficial effects for patients by addressing the range of different substrates and effects observed in MLIII. This is the first time that this type of analysis has been conducted for patients with MLIII. The results can also be extrapolated to other inborn errors of metabolism.

## References

[B1] Alegra T, Sperb-Ludwig F, Guarany NR, Ribeiro EM, Lourenço CM, Kim CA, Valadares ER, Galera MF, Acosta AX, Horovitz DDG (2019). Clinical characterization of Mucolipidosis II and III: A multicentric study. J Pediatr Genet.

[B2] Asatsuma-Okumura T, Ito T, Handa H (2020). Molecular mechanisms of the teratogenic effects of thalidomide. Pharmaceuticals (Basel).

[B3] Braulke T, Bonifacino JS (2009). Sorting of lysosomal proteins. Biochim Biophys Acta.

[B4] Bosch ME, Kielian T (2015). Neuroinflammatory paradigms in lysosomal storage diseases. Front Neurosci.

[B5] Boutry M, Branchu J, Lustremant C, Pujol C, Pernelle J, Matusiak R, Seyer A, Poirel M, Chu-Van E, Pierga A (2018). Inhibition of lysosome membrane recycling causes accumulation of gangliosides that contribute to neurodegeneration. Cell Rep.

[B6] Breiden B, Sandhoff K (2020). Mechanism of secondary ganglioside and lipid accumulation in lysosomal disease. Int J Mol Sci.

[B7] Coutinho MF, Santos JI, Alves S (2016). Less is more: Substrate reduction therapy for lysosomal storage disorders. Intern J Molec Scien.

[B8] Cox TM (2005). Substrate reduction therapy for lysosomal storage diseases. Acta Pediatr.

[B9] Elliot-Smith E, Speak A, Lloyd-Evans E, Smith DA, Spoel AC, Jeyakumar M, Butters TD, Dwek RA, d’Azzo A, Platt FA (2008). Beneficial effects of substrate reduction therapy in a mouse model of GM1 gangliosidosis. Mol Genet Metab.

[B10] Guémy C, Laforêt P (2023). The new horizons for treatment of Late-Onset Pompe Disease (LOPD). Revue Neurol (Paris).

[B11] Hasilik A, Neufeld EF (1980). Biosynthesis of lysosomal enzymes in fibroblasts. Synthesis as precursors of higher molecular weight. J Biol Chem.

[B12] Hasilik A, Von Figura K (1981). Oligosaccharides in lysosomal enzymes. Distribution of high-mannose and complex oligosaccharides in cathepsin D and beta-hexosaminidase. Eur J Biochem.

[B13] Ito T, Handa H (2020). Molecular mechanisms of thalidomide and its derivatives. Proc Jpn Acad Ser B Phys Biol Sci.

[B14] Jung YJ, Tweedie D, Scerba MT, Greig NH (2019). Neuroinflammation as a factor of neurodegenerative disease: Thalidomide analogs as treatments. Front Cell Dev Biol.

[B15] Kavanagh K, Pastores GM (2021). Hepatic manifestations of lysosomal storage disorders: Differential diagnosis, investigations, and treatment, current and upcoming. EMJ.

[B16] Khan SA, Tomatsu AC (2020). Mucolipidoses overview: Past, present, and future. Int J Mol Sci.

[B17] Kornfeld S, Sly WS, Scriver CR, Beaudet al -, Sly WS, Valle D (2001). The Metabolic and Molecular Bases of Inherited Disease.

[B18] Masingue M, Dufour L, Lenglet T, Saleille L, Goizet C, Ayrignac X, Ory-Magne F, Barth M, Lamari F, Mandia D (2020). Natural history of adult patients with GM2 gangliosidosis. Ann Neurol.

[B19] Meikle PJ, Hopwood JJ, Clague AE, Carey WF (1999). Prevalence of lysosomal storage disorders. JAMA.

[B20] Mukund V (2020). Genistein: Its role in breast cancer growth and metastasis. Curr Drug Metab.

[B21] Okada S, Owada M, Sakiyama T, Yutaka T, Ogawa M (1985). I-cell disease: Clinical studies of 21 Japanese cases. Clin Genet.

[B22] Otomo T, Hossain MA, Ozono K, Sakai N (2012). Genistein reduces heparan sulfate accumulation in human mucolipidosis II skin fibroblastos. Mol Genet Metab.

[B23] Papadopoulos C, Meyer H (2017). Detection and clearance of damaged lysosomes by the endo-lysosomal damage response and lysophagy. Curr Biol.

[B24] Patterson MC, Vecchio D, Prady H, Abel L, Wraith JE (2007). Miglustat for treatment of Niemann-Pick C disease: A randomized controlled study. Lancet Neurol.

[B25] Pineda M, Walterfang M, Patterson MC (2018). Miglustat in Niemann-Pick disease type C patients: A review. Orphanet J Rare Dis.

[B26] Piotrowska E, Jakobkiewicz-Banecka J, Tylki-Szymanska A, Liberek A, Maryniak A, Malinowska M, Czartoryska B, Puk E, Kloska A, Liberek T (2008). Genistin-rich soy isoflavone extract in substrate reduction therapy for Sanfilippo syndrome: An open-label, pilot study in 10 pediatric patients. Curr Ther Res.

[B27] Reitman ML, Varki A, Kornfeld S (1981). Fibroblasts from patients with I-cell disease and pseudo-Hurler polydystrophy are deficient in uridine 5’-disphosphate-N-acetylglucosamine: glycoprotein-N-acetylglucosaminylphosphotransferase activity. J Clin Invest.

[B28] Sato T, Gotoh M, Kiyohara K, Akashima T, Iwasaki H, Kameyama A, Mochizuki H, Yada T, Inaba N, Togayachi A (2003). Differential roles of two N-acetylgalactosaminyltransferases, CSGalNAcT-1, and a novel enzyme, CSGalNAcT-2. J Biol Chem.

[B29] Seo CH, Kim JB (2015). Therapeutic potential of resveratrol in type I Gaucher disease. Phytother Res.

[B30] Simonaro CM (2016). Lysosomes, lysosomal storage diseases, and inflammation. J Inborn Errors Metab Screen.

[B31] Tiede S, Cantz M, Spranger J, Braulke T (2006). Missense mutation in the N-acetylglucosamine-1-phosphotransferase gene (GNPTA) in a patient with mucolipidosis II induces changes in the size and cellular distribution of GNPTG. Hum Mutat.

[B32] Walkley SU (2009). Pathogenic Cascades in Lysosomal Disease - Why so Complex?. J Inherit Metab Dis.

[B33] Yoo S, Kim JB (2015). Anti-apoptotic and beneficial metabolic activities of resveratrol in type II Gaucher disease. Biol Pharm Bull.

[B34] Zhu SY, Yao RQ, Li YX, Zhao PY, Ren C, Du XH, Yao YM (2020). Lysosomal quality control of cell fate: A novel therapeutic target for human diseases. Cell Death Dis.

